# Management of dental extraction in patients with Haemophilia A and B: A report of 58 extractions

**DOI:** 10.4317/medoral.19191

**Published:** 2013-10-13

**Authors:** Andre Peisker, Gregor F. Raschke, Stefan Schultze-Mosgau

**Affiliations:** 1 DMD, Department of Cranio-Maxillofacial & Plastic Surgery, Friedrich Schiller University Jena, Erlanger Allee 101, 07747 Jena, Germany; 2 MD, DMD, Department of Cranio-Maxillofacial & Plastic Surgery, Friedrich Schiller University Jena, Erlanger Allee 101, 07747 Jena, Germany; 3 MD, DMD, PhD, Department of Cranio-Maxillofacial & Plastic Surgery, Friedrich Schiller University Jena, Erlanger Allee 101, 07747 Jena, Germany

## Abstract

Objectives: Patients with inherited bleeding disorders are at high risk of bleeding following oral surgery and present challenges to the oral surgeons. Aim of this study was to report our experience in dental extraction in patients exhibiting Haemophilia A and B between 2007 and 2012. Patient and Methods: 58 dental extractions in 15 patients during 19 interventions were performed. Replacement therapy with recombinant and plasma-derived factor VIII and IX was applied systematically in combination with antifibrinolytic treatment and local haemostatic measures. The following data were recorded: type of surgery, applied local haemostatic measures, general substitution, systemic antifibrinolytic agents and occurrence of postoperative bleeding complications. Results: Two patients presented postoperative bleeding. One had secondary bleeding requiring additional injection of factor concentrates. The other one presented epistaxis which was managed conservatively with a nasal tamponade. Conclusions: Excellent haemostasis is achievable after dental extractions in patients with Haemophilia A and B by following a protocol using defined pre- and postoperative doses of factor concentrates in combination with haemostatic measures.

** Key words:**Antifibrinolytic treatment, dental extraction, Haemophilia, inherited bleeding disorders, local haemostatic measures.

## Introduction

Haemophilia is a common hereditary bleeding disorder. More than 400,000 people are affected worldwide (1). The X-linked recessive chromosomal bleeding disorder is caused by a variety of mutations in the factor VIII (Haemophilia A) or factor IX (Haemophilia B) gene. As a result men are expressing the disease, meanwhile women are typically asymptomatic carriers. One third of all cases of Haemophilia are the result of spontaneous mutations. Two thirds have a prior family history.

Haemophilia A is the most common form of this disorder. Approximately 1:5,000 males are affected. In comparison Haemophilia B is less often, 1:30,000 males are affected (2). Haemophilia is summarized as follows: mild when plasma activity is between 6 and 40 % of normal; moderate if it ranges between 1-5 % and severe if it is <1 % (3).

Patients with Haemophilia are at high risk of intra- and postoperative bleeding when oral surgery has to be performed. Therefore management of patients with hereditary bleeding disorders requires close cooperation between oral surgeons and a comprehensive Haemophilia treatment center. The use of clotting factor replacement therapy for all invasive surgical interventions is required (4,5). Successful treatment protocols are described in the current literature using systemic treatment, antifibrinolytic agents and local haemostatic measures (6,7).

Purpose of the presented study was to report our experience in dental extractions and to evaluate the efficacy of a systematic protocol involving systemic treatment combined with local haemostatic techniques to prevent bleeding after dental extraction in patients with Haemophilia.

## Patient and Methods

The study includes results of 58 extractions in 15 patients. General anesthesia was performed if necessary. Articain with Adrenaline 1:100,000 (Ultracain D-S forte, Sanofi-Aventis, Paris, France) was used for local anesthesia. Recombinant and plasma-derived factor VIII and IX were applied for systemic treatment. The selected factor replacement therapy protocols are given in table 1. The following formulas were applied for calculating the factor dose:

Dosage (units) = body weight (kg) × desired factor VIII rise (IU/dL or % of normal) × 0.5 and Dosage (units) = body weight (kg) × desired factor IX rise (IU/dL or % of normal).

The replacement with doses of native plasma derived factor VIII was initiated in four patients exhibiting severe Haemophilia A and two patients with mild Haemophilia A. Doses of recombinant factor VIII were used in two patients with severe Haemophilia A and two patients with mild Haemophilia A. In one patient with mild Haemophilia A, treatment with Desmopressin (DDAVP) increased satisfying the factor VIII level without factor concentrate injection. Two weeks earlier a prior testing had been carried out with Minirin to assess the level of patient response.

Doses of native plasma derived factor IX were used in three patients exhibiting severe Haemophila B and one patient with moderate Haemophilia B.

In all cases injection was administered half-hour before surgery. The clotting factor level was planned to increase from 50-100 IU/dL of normal preoperatively. The postoperative factor trough levels were aimed to remain above 30 % ( Table 1). Further doses of clotting factor were applied in the next days.

**Table 1:**
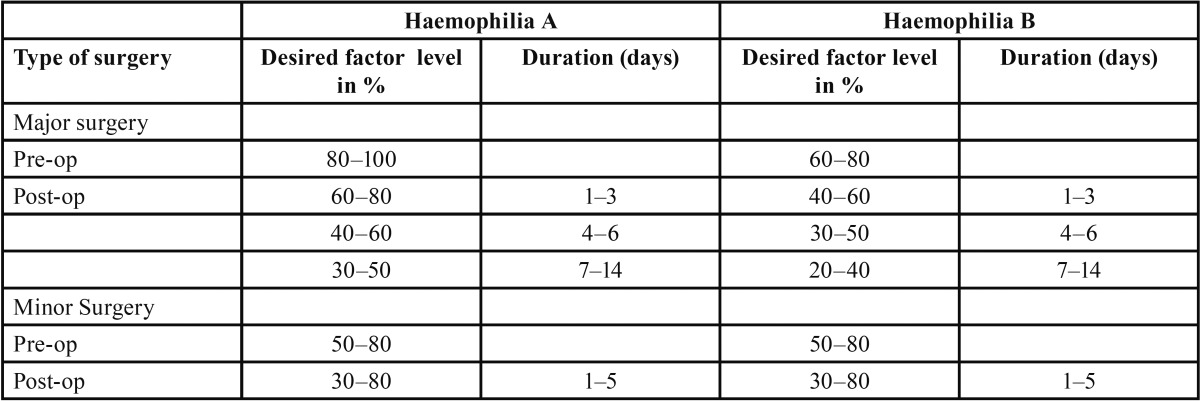
Plasma factor level and duration of the replacement therapy needed for surgical interventions in patients with Haemophilia (23).

Tranexamic acid (Cyklokapron, Meda Pharma, Bad Homburg, Germany) was used systematically in 16 interventions. It was administered intravenous and orally at a dose of 20 mg/kg of body weight every eight hours for seven days. Due to cardiovascular contraindications it was not used in three cases.

Furthermore gauzes saturated with tranexamic acid were applied in all patients during the first three days after surgery. Patients with contraindications for systemic antifibrinolytic agents received tranexamic acid only topical.

Local haemostatic measures were applied in all cases according to surgeon preference and practice pattern ( Table 2). Collagen vlies (Lyostypt, B. Braun Melsungen, Melsungen, Germany), oxycellulose (Tabotamp, Ethicon Johnson&Johnson, New Brunswick, USA), fibrin glue (Tissucol Duo S, Baxter, Munich, Germany) resorbable suture (Vicryl, Ethicon Johnson&Johnson, New Brunswick, USA) were used.

**Table 2:**
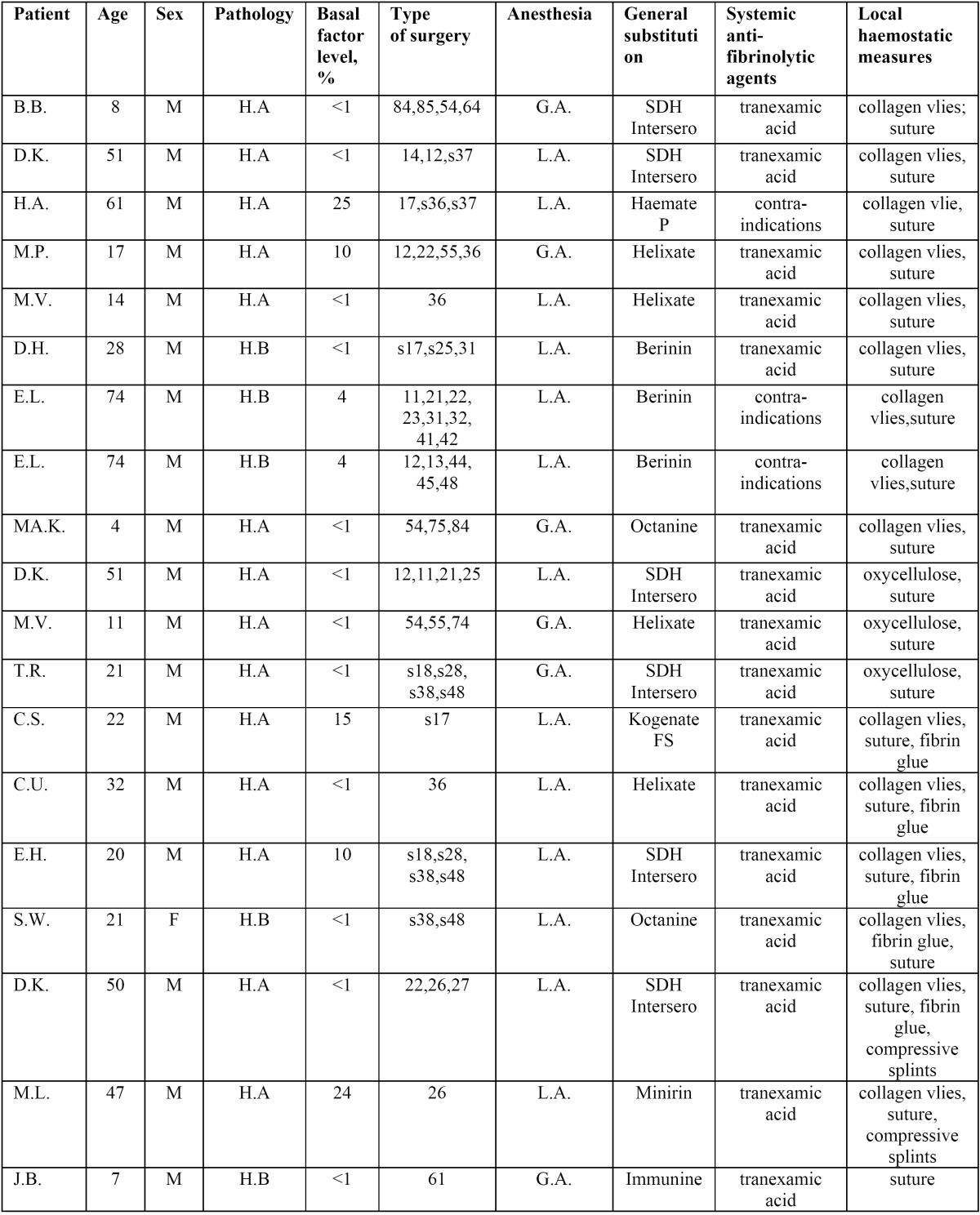
Distribution of patients included in this report: type of surgery, anesthesia, substitution and local haemostatic measures.

Antibiotics were prescribed if clinically necessary. Postoperative pain was controlled by adequate doses of Paracetamol.

## Results

Distribution of patients is given in  Table 2. Overall 58 dental extraction's in 15 patients aged 4 to 74 years, mean age 32 ± 22.5 years (mean ±SD), 14 male (93%), one female (7%) during 19 interventions over a period of 6 years (2007-2012) were performed. 11 patients (73%) were affected by Haemophilia A (six from the severe and five from the moderate form) and four patients (27%) by Haemophilia B (three from the severe and one from the mild form).

General anesthesia was necessary in six cases and local anesthesia in 13 cases. Secondary bleeding occurred in two patients with mild Haemophilia A. First patient, who had had all four wisdom teeth removed in local anesthesia, bled from the nose on the seventh postoperative day which was controlled with a nasal tamponade left in situ for two days. Second patient, who had had three teeth extracted, bled on the fifth day after surgery. To control bleeding this case required the additional use of clotting factor replacement therapy and repeated compression with gauzes saturated with tranexamic acid. No general complications were noted. All patients were hospitalized for a mean period of six days.

## Discussion

Patients with Haemophilia are at high risk of secondary bleeding following oral surgery. Close cooperation between haematologists and oral surgeons is necessary to prevent excessive haemorrhage. International guidelines strongly recommend the use of clotting factor replacement therapy for all invasive surgical interventions (5). The World Federation of Haemophilia (WFH) ad-vises the use of factor concentrates to cryoprecipitate or fresh frozen plasma for the replacement therapy in patients with Haemophilia. Factor VIII and IX concentrates may be divided into two categories, recombinant and plasma-derived factor. There are no statements from the WFH for a preference for recombinant over plasma-derived concentrates. The level of factor required for surgical procedures in haemophiliacs is given in  Table 1. In this study we used native plasma derived concentrates in 10 cases and recombinant concentrates in four cases. One unit of infused recombinant factor VIII per kilogram of body weight should lead to a rise of plasma factor VIII level of approximately 2% in patients with Haemophilia A and in absence of an inhibitor. The half-life of factor VIII is approximately 8–12 h. On the other side, one unit of infused plasma-derived concentrates of factor IX per kilogram of body weight will raise the plasma factor IX activity by approximately 1% in patients with Haemophilia B in absence of an inhibitor (8). In contrast to the native plasma-derived factor, the recombinant factor IX has a lower recovery. One unit of infused factor IX per kg body weight should raise the plasma factor IX level approximately 0.8% in adults and 0.7 % in children (9). The half-life of factor IX is approximately 18–24 h (10).

The conventional replacement therapy is not effective in patients with antibodies to factor VIII and IX. Approximately 8 to 20% of the patients with severe Haemophilia A and 2.5 to 16% of those with severe Haemophilia B are affected (11). A variety of genetic and immunologic factors are responsible in inhibitor development (12-16). However an activated prothrombin complex concentrate or a recombinant activated factor VII concentrate can be used to this patients (17).

Factor replacement therapy carries a risk of transmission of viruses or new pathogens and may lead to develop inhibitors. Therefore reducing the number of patients and times requiring factor replacement therapy is helpful. DDAVP may be the treatment of choice for patients with mild or moderate Haemophilia A (18,19). A preoperative single dose of 0.3 µg/kg of body weight can increase the factor VIII level three- to sixfold in good responders (20). It is much less expensive than coagulation factor concentrates. There is no risk of transmission of infections and no risk of immunization. Because of the significant differences between individuals, a prior testing of patient’s response a few weeks before surgery is necessary in these cases. Various reports have described dental extractions under DDAVP in patients with bleeding disorders (21,22).

The antifibrinolytic treatment with tranexamic acid prevents postoperative bleeding by inhibiting the activation of plasminogen to plasmin and promoting the clot stability. It is usually given as an oral tablet three to four times or by intravenous infusion two to three times daily. Tranexamic acid should be prescribed for seven days following dental extractions in patients with intrinsic bleeding disorders (23). Furthermore various reports referred the successful local use of tranexamic acid for the reduction of oral bleeding (22,24).

Oral surgeons have to use all techniques to minimise the probability of intra- and postoperative bleeding. Local haemostatic measures are obligate following dental extraction in these patients. Typical agents are sutures (22), collagen vlies (21), oxycellu-lose (25), gelatine (26), a fibrin glue (27) and cyanoacrylate (28). In this study all mentioned local measures were used according to surgeon preference and practice pattern. Furthermore the different agents were combined with each other to potentiate the haemostatic effect. Wagner et al. compared often used topical haemostatic agents in terms of their ability to mediate platelet aggregation, deposition and activation in a series of in vitro tests. He presented an overall activity ranking of the materials used: collagen>gelatine>oxidized regenerated cellulose (29). In this report we could not note a different efficacy between the various local haemostatic measures. To prevent late bleeding we used absorbable sutures in order to avoid suture removal. For pain control non-steroidal anti-inflammatory drugs and aspirin which affect platelet function must be avoided. Paracetamol is a safe alternative to prevent postoperative pain.

The current literature reports successful treatment protocols to prevent bleeding complications following oral surgery procedures. The results are similar to our report. Frachon et al. evaluated the efficacy of a protocol using a general management and combining fibrin glue with gelatin packing and compressive splints for the management of dental extractions in haemophiliacs. An outcome with six instances of postsurgical bleeding during 19 interventions was recorded (22). Frachini et al. analysed 10 years of experience in managing patients with hereditary bleeding disorders in Italy. No specific protocol was used. Only 10 bleeding complications (1.9 %) were reported when patients are managed by local and systemic measures (24). Piot et al. reported also good results for dental extractions in patients with bleeding disorders. Collagen vlies, fibrin suture and primary suture were used in combination. They reported two cases of secondary bleeding during 103 dental extractions in 93 patients (21). Zanon et al. registered two bleeding complications after dental extraction in 75 procedures. Clotting factor concentrates to obtain a plasma peak level of 30% were given. Fibrin sponges, primary suture and tranexamic acid were used for local measures (30).

In our study sample secondary bleeding occurred in two patients surprisingly exhibiting mild form of Haemophilia A. This is an indication that postoperative bleeding after oral surgery in patients exhibiting Haemophilia is difficult to predict also if defined treatment protocols are used. Postoperative monitoring of these patients is necessary to prevent excessive haemorrhage.

## Conclusion

Considering an individual haemostaseological protocol in consultation with a hematologist helps to prevent bleeding complications in haemophiliacs undergoing dental extractions. Local haemostatic measures have to be used whenever possible following a dental extraction to reduce the need of factor concentrates. A specialist dental service in consultation with a Haemophilia treatment centre should perform these procedures.
